# Characterization of Genes Encoding for Acquired Bacitracin Resistance in *Clostridium perfringens*

**DOI:** 10.1371/journal.pone.0044449

**Published:** 2012-09-06

**Authors:** Audrey Charlebois, Louis-Alexandre Jalbert, Josée Harel, Luke Masson, Marie Archambault

**Affiliations:** 1 Department of pathology and microbiology, Faculty of Veterinary Medicine of the University of Montreal, Centre de Recherche en Infectiologie Porcine (CRIP), Saint-Hyacinthe, Québec, Canada; 2 Biotechnology Research Institute, Montréal, Québec, Canada; The University of Hong Kong, Hong Kong

## Abstract

Phenotypic bacitracin resistance has been reported in *Clostridium perfringens*. However, the genes responsible for the resistance have not yet been characterized. Ninety-nine *C. perfringens* isolates recovered from broilers and turkeys were tested for phenotypic bacitracin resistance. Bacitracin MIC_90_ (>256 µg/ml) was identical for both turkey and chicken isolates; whereas MIC_50_ was higher in turkey isolates (6 µg/ml) than in chicken isolates (3 µg/ml). Twenty-four of the 99 isolates showed high-level bacitracin resistance (MIC breakpoint >256 µg/ml) and the genes encoding for this resistance were characterized in *C. perfringens* c1261_A strain using primer walking. Sequence analysis and percentages of amino acid identity revealed putative genes encoding for both an ABC transporter and an overproduced undecaprenol kinase in *C. perfringens* c1261_A strain. These two mechanisms were shown to be both encoded by the putative *bcrABD* operon under the control of a regulatory gene, *bcrR*. Efflux pump inhibitor thioridazine was shown to increase significantly the susceptibility of strain c1261_A to bacitracin. Upstream and downstream from the *bcr* cluster was an IS*1216-*like element, which may play a role in the dissemination of this resistance determinant. Pulsed-field gel electrophoresis with prior double digestion with I-CeuI/MluI enzymes followed by hybridization analyses revealed that the bacitracin resistance genes *bcrABDR* were located on the chromosome. Semi-quantitative RT-PCR demonstrated that this gene cluster is expressed under bacitracin stress. Microarray analysis revealed the presence of these genes in all bacitracin resistant strains. This study reports the discovery of genes encoding for a putative ABC transporter and an overproduced undecaprenol kinase associated with high-level bacitracin resistance in *C. perfringens* isolates from turkeys and broiler chickens.

## Introduction

*Clostridium perfringens* is a Gram-positive, anaerobic spore-forming bacterium that causes a wide variety of diseases in humans and animals. A classification based on the production of four major toxins (alpha, beta, epsilon, and iota) divides the *C. perfringens* into five toxigenic biotypes (A to E) [1]. This microorganism is a commensal of the gastrointestinal tract of mammals. It is also commonly found in soil and water [2]. *C. perfringens* is responsible for gas gangrene, enteritis necroticans, food poisoning, and non-foodborne gastrointestinal infections in humans [3]. It is also associated with a variety of enteric diseases in many animal species [4]. Isolates of animal origin constitute a risk for transmission to humans through the food chain. *C. perfringens* has particular significance in poultry where it may cause necrotic enteritis [5]. The disease cost to the international poultry industry has been estimated to be above $US2 billion per year [6].

Necrotic enteritis is usually controlled by the addition of bacitracin antimicrobial, a polypeptide antibiotic synthesized by *Bacillus licheniformis* and some strains of *Bacillus subtilis,* in feed. It inhibits cell wall synthesis by binding to undecaprenyl pyrophosphate (UPP) and preventing its dephosphorylation, thereby blocking the recycling of UPP to undecaprenol monophosphate (UP also known as C_55_-P transporter), a lipid carrier implicated in the transport of peptidoglycan monomer units through the cytoplasmic membrane [7], [8]. In humans, bacitracin is currently used topically in therapy and has been proposed and tested as an oral drug for the control of vancomycin-resistant enterococci with limited success [9]. This antibiotic is nephrotoxic when administered systematically [10]. However, oral therapy is reported to be safer as the drug is not significantly absorbed by the gastrointestinal tract [11]. In animals, bacitracin is largely used in therapy and for prophylaxis purposes. Although banned in Europe as a growth promoter since 1999, bacitracin is still used as a feed additive in some countries namely, Canada, New Zealand and the United States [12], [13], [14].

To date, four major bacitracin resistance mechanisms have been described. The *bacA* gene encodes for an undecaprenyl pyrophosphate phosphatase (UppP) and was discovered in 1992 in *Escherichia coli* [15] and renamed *uppP* in 2004 [16]. This enzyme generates UP from UPP [16]. Homologues to UppP have been described in *Streptococcus pneumonia*, *Staphylococcus aureus* and *Enterococcus faecalis* V583 [17]. The *bcrABC* genes were identified in 1995 in *Bacillus licheniformis* [18]. These genes encode for an ABC transporter that pumps the bacitracin molecule out of the bacterial cell. Homologues to this transporter have been found in *B. subtilis*, *Streptococcus mutans* and *E. faecalis* [19]. Another mechanism is the overproduction of an undecaprenol kinase which converts undecaprenol to UP, increasing the amount of lipid carrier in the bacterial cell [20]. The *bcrD* gene of *E. faecalis* identified by Manson et al. [21] encodes for an undecaprenol kinase. It was found to have significant sequence identity to a putative undecaprenol kinase in *Clostridium thermocellum*. The last mechanism involves exopolysaccharide molecules. In *Xanthomonas campestris*, *Sphingomonas* strains S-88 and NW11, and *E. coli* K-12 [22], [23] different mutations that inhibit the synthesis of exopolysaccharides have been associated with bacitracin resistance. It is believed that the synthesis of these non-essential polymers also requires the UP transporter, and these mutations therefore indirectly provide an increased supply of this transporter for the synthesis of the essential cell wall component, the peptidoglycan [16]. For *S. mutans,* it is speculated that rhamnose-glucose polysaccharide may act as a barrier that prevents some antibiotics from reaching their targets [24].

In *E. faecalis*, an ABC transporter and an overproduced undecaprenol kinase were both reported to be encoded by the *bcrABD* operon under the control of a regulator, *bcrR* [21]. This regulator has been described as a membrane bound sensor and a transducer of bacitracin availability to regulate *bcrABD* expression [21]. This gene cluster was reported to be located on a transferable plasmid [21]. In *E. faecalis*, these genes have been associated with high-level bacitracin resistance with a minimum inhibitory concentration (MIC) >256 mg/L [25]. It was recently found that the BcrAB transporter was sufficient to confer high-level bacitracin resistance in *E. faecalis* [25].

Despite studies on bacitracin resistance in *C. perfringens* in North America which show high levels of resistance [26], [27], [28], the genes responsible for the resistance have not yet been identified. In this study, we report the discovery of putative genes encoding for an ABC transporter, an overproduced undecaprenol kinase and a regulatory protein associated with high-level bacitracin resistance in *C. perfringens* isolates from turkeys and broiler chickens. We also demonstrate that *bcrABDR* genes are located on the chromosome.

## Materials and Methods

### Ethics Statement

The handling of animals of this study was performed in accordance with current national Canadian legislation (Meat Inspection and Health of Animals Acts of Canada No. SOR/90–288) under the enforcement of the Canadian Food Inspection Agency (CFIA) inspectors. All fecal samples of this study were taken from already slaughtered animals in federally registered slaughter facilities where federal measures are in place to protect food animals during handling and slaughter.

### Bacterial Isolates

Isolates of *C. perfringens* from conventionally raised birds were recovered from the normal intestinal microbiota of chicken and turkey taken at five (four chicken and one turkey) processing plants (Berthierville, Saint-Damase I and II, Saint-Anselme, and Saint-Jean-Baptiste) located in the province of Quebec, Canada. Only three isolates were associated with necrotic enteritis. Antimicrobial regimens were not available. The contents of the caeca were cultured in cooked meat broth (PML, Québec, Canada) for 48 h in anaerobic conditions at 35°C. Ten µl were then plated on anaerobic sheep blood agar (PML) and incubated for 24 h under anaerobic conditions. Three greyish colonies with a typical double zone of hemolysis were subcultured for purity on blood agar. Gram staining was performed on every isolate and cultures with Gram-positive square-ended rods were selected for PCR identification and genotyping. One to two *C. perfringens* isolates per bird were kept for this study.

### PCR Identification and Genotyping

A multiplex PCR was used for the detection of the toxin genes *cpa* (alpha), *cpb* (beta), *etx* (epsilon), *iA* (iota), *cpb2* (β2- toxin) and *cpe* (enterotoxin) of *C. perfringens*. Primers and conditions used were as previously described [29] with slight modifications to allow smaller sample and reaction volumes. DNA extraction was performed with the Chelex 100 (Bio-Rad, Mississauga, Ontario, Canada) ebullition method where many loops of pure colonies of the isolates were mixed with 10% Chelex then boiled for 20 min [30]. The supernatant contains the DNA used for the multiplex PCR. Each PCR reaction was constituted of: 2.5 µl of 10X PCR Buffer (Ge Healthcare, Québec, Canada), 1.5 U of Taq DNA polymerase (Ge Healthcare) and primer sets for *cpa*, *cpb*, *etx*, *iA*, *cpe*, and *cpb2*. DNA amplification reactions were carried out using a Whatman Biometra thermocycler (Montreal Biotech Inc, Québec, Canada) programmed as follows: 35 cycles of 20 sec at 94°C, 20 sec at 55°C and 40 sec at 72°C with a hot start of 5 min at 94°C and a final elongation time of 5 min at 72°C. For visualization, 5 µl of the PCR reaction were subjected to electrophoresis in 1% agarose gel stained with ethidium bromide. A 100 bp ladder (TrackIt, Invitrogen, Ontario, Canada) was used as a marker. *C. perfringens* type E (AHL#155, positive for *cpa*, *iA*, *cpe* and *cpb2* genes) and *C. perfringens* type B (AHL#156, positive for *cpa*, *cpb* and *etx* genes) [28] were used as positive controls.

### Multiple-locus Variable Number of Tandem Repeats Analysis

Multiple-locus variable number of tandem repeats analysis (MLVA) was performed as previously described by Chalmers et al. [31] on all bacitracin resistant strains. The PCR products were analysed in 2% agarose gel stained with ethidium bromide. A TrackIt 100 bp ladder (Invitrogen) was used as a marker. MLVA profiles, created from the number of repeats for each of the VNTR loci, were imported to the BioNumerics software (Applied Maths, Texas, USA). The unweighted pair group method with arithmetic mean (UPGMA) was used for clustering. Band position tolerance and optimisation were set to 1%. Identity cut-off was set at 97%.

### Bacitracin Susceptibility Testing

Bacitracin minimum inhibitory concentrations (MICs) were determined using the Etest (AB BIODISK, Solna, Sweden) according to the manufacturer’s instructions. Preliminary tests indicated that an inoculum size of 10^5 ^CFU/ml allowed the growth of a lawn of well-developed colonies and an easily permitted discernment of growth inhibition by bacitracin. Consequently, an inoculum of 10^5^ CFU/ml was used for evaluation of bacitracin susceptibilities. Stock cultures were thawed then plated on blood agar plates and incubated for 24 h in anaerobic conditions. Colonies were randomly selected, combined and then diluted in Brucella broth to yield concentrations of 10^5^ CFU/ml. *C. perfringens* suspensions were spread with a cotton swab on a Brucella blood agar plate. After the surface of the inoculated plate had completely dried, a bacitracin Etest strip was placed on the surface according to the manufacturer’s instructions. Agar plates were then incubated at 35°C in anaerobic conditions for 48 h with a first reading at 24 h and a second reading at 48 h. At both times, colonies could be detected and MICs were read by determining where the zone of growth inhibition intersected the MIC scale on the strip. *S. aureus* ATCC 29213 and *C. perfringens* ATCC 3110 were used as quality controls. The concentrations that inhibited growth of at least 50% and 90% of the isolates tested were calculated as MIC_50_ and MIC_90_, respectively. Low level bacitracin resistance breakpoint was set at >16 µg/ml while high-level bacitracin resistance was set at >256 ug/ml according to previous studies [21], [31].

### Detection of Bacitracin Resistance Genes and Sequencing

Sequencing of the *bcr* operon was performed on a bacitracin resistant *C. perfringens* strain c1261_A isolated from the normal flora of a turkey caecum using the primer walking technique. Primers for the *bcrR* and *bcrB* genes (see Table S1 in the supplemental material) were both designed based on the *E. faecalis* sequences of these genes [21]. Both *bcrR* and *bcrB* genes were detected by PCR in *C. perfringens* strain c_1261A. Amplicons were purified by Qiaquick PCR purification kit (Qiagen, Mississauga, Canada), sequenced on an ABI PRISM 310 (Applied Biosystems), and results were used for primer walking. Briefly, the targeted sequences were further divided in different section for which primer pairs were designed for PCR and sequencing (Table S1). Each section was amplified using a Whatman Biometra thermocycler (Montreal Biotech Inc, Québec, Canada) programmed as follows: 35 cycles of 10 sec at 94°C, 10 sec at 50°C and 30 sec at 72°C with a hot start of 5 min at 94°C and a final elongation time of 5 min at 72°C. Each amplicon was purified and sequenced. Sequence alignment was performed using BioEdit Sequence Alignment Editor (Ibis Biosciences) and sequence analysis was done with the BLASTN and BLASTP programs (National Center for Biotechnology Information, Los Alamos, New Mexico). GeneMark.hmm software was used to locate the gene boundaries [32]. New primers for *bcrA*, *bcrB*, *bcrD* and *bcrR* were designed based on the novel sequences. Bacitracin resistant *C. perfringens* isolates were tested by PCR for the presence of these genes. The PCR mixture contained 1X PCR buffer, 200 µM of dNTPs, 320 nM of each primer (Table S1), 1 unit of Taq DNA polymerase (New England Biolabs, Ontario, Canada), and 5 µL of the DNA template in a total volume of 25 µl. The conditions were: 30 cycles of 1 min at 94°C, 1 min at 55°C and 1 min at 72°C with a hot start of 1 min at 94°C and a final elongation time of 1 min at 72°C. A volume of 10 µl of each PCR product was separated for 25 min at 150 V on a 1.5% agarose gel stained with ethidium bromide. To positively correlate the absence of a complete *bcr* locus with phenotypic susceptibility to bacitracin, 70 susceptible *C. perfringens* strains of this study were analyzed for the presence or absence of *bcrABDR* genes. This screening was performed on 70 of the 75 susceptible isolates collected in this study because five of those were not able to grow on subculture for this experiment.

### Microarray

Total genomic DNA extracted from bacitracin resistant isolates were analyzed by a custom DNA microarray as previously described [33], with a few modifications. Briefly, we have previously designed and validated antimicrobial resistance microarrays for use with both Gram-negative and Gram-positive bacteria [34], [35], [36] using a 70-mer probe length to maximize the overall sensitivity of the microarray [37]. A total of 173 probes were used, including 166 antimicrobial resistance genes and a class 1 integron. Sixty-five oligonucleotide probes printed on the microarray were selected directly from a previous study [38]. This array was supplemented with new probes [39], [40] found in Table S2 in the supplemental material. DNA was labelled using the Bioprime DNA labeling system (Invitrogen) and purified using PureLink PCR purification kit (Invitrogen) according to the manufacturer’s protocol. Labelled DNA was then hybridized on the antimicrobial resistance microarrays which were then scanned by a scanarray express microarray scanner (Packard Biosciences, Billerica, MA, USA). Bacitracin susceptible *C. perfringens* ATCC 13124 was used as a control.

### Efflux Pump Inhibitors

The effect of (ABC) efflux pump inhibitors on bacitracin susceptibility was evaluated by a checkerboard microdilution technique as previously described by Hendricks et al. [41]. Inhibitors tested were thioridazine, prochlorperazine, reserpine, verapamil, MK-571, and probenecid (Sigma-Aldrich, Oakville, Ontario, Canada). Combinations of concentrations of inhibitors (0–2048 µg/ml) and bacitracin (0–512 µg/ml) were added to the plates in order to investigate the influence of the tested inhibitors on the MIC values against resistant strain c1261_A. Three independent checkerboard assays were performed.

### Plasmid Analysis

Plasmid DNA of the bacitracin resistant *C. perfringens* strain c1261_A was purified by an alkaline lysis method using a plasmid midi-kit (Qiagen) with the addition of 5 µg/ml of lysozyme in the resuspension buffer before incubation at 35°C for 30 min. The plasmid DNA was visualized on a 1% agarose gel after an electrophoresis of 60 min at 100 V. The plasmid bands sizes were determined using the software 1Dscan EX 3.01 (BD Biosciences BioImaging) of the Gel Doc 2000 (Bio Rad) by reference to plasmids of known band sizes (Bac-Tracker Supercoiled DNA ladder, Epicentre Biotechnologies). Plasmid curing experiments were also performed as previously described [42] using mitomycin C or the combination of acridine orange and elevated temperature.

### PFGE Analysis

All genomic DNAs were prepared as described previously [43]. DNA plugs were double-digested with 40 U of I-CeuI (New England Biolabs) and 80 U of MluI (New England Biolabs) for 1 h at 37°C. The I-CeuI restriction enzyme cuts only in the 23S rRNA genes (*rrn* genes), which are localized only on the chromosome. Thus, chromosomal fragments can be distinguished from plasmid bands with the *rrn* probe. DNA fragments were then separated by contour-clamped homogenous electric field (CHEF) electrophoresis with the following conditions: migration of 16 h at 15°C in a 0.8% agarose gel, with a voltage of 6 V/cm and switch times of 0.5 to 40 s. A low range PFG marker (New England Biolabs) was used as a DNA ladder. Gels were stained with ethidium bromide and bands were visualized with the software 1Dscan EX 3.01 (BD Biosciences BioImaging) of the Gel Doc 2000 (Bio Rad).

### Southern Hybridization Analysis

The PFGE genomic DNAs and plasmid extractions were transferred to positively charged nylon membranes (Roche Diagnostics, Laval, Québec, Canada) using a Vacuum Blotter Model 785 (Bio-Rad). Amplicons derived from the *bcrB* and *rrn* genes were used as hybridization probes. The PCR products were labelled with digoxigenin using the PCR DIG probe synthesis kit (Roche Diagnostics) and were detected with the DIG nucleic acid detection kit (Roche Diagnostics), both according to the manufacturer’s instructions.

### RNA Isolation and Semi-quantitative RT-PCR

Total *C. perfringens* c1261_A RNA was extracted with the RNeasy mini kit (Qiagen) from 1 ml of an overnight BHI culture supplemented with 0, 10, 50, 100 or 256 µg/mL of bacitracin. RNA was quantified and stored −80°C. Two µg of RNA was converted to cDNA using the Quantitect reverse transcriptase kit (Qiagen) according to the manufacturer’s instructions. PCR for the genes *bcrA*, *bcrB*, *bcrD* and *bcrR* were done on the same amount of cDNA from each condition. 16 s rRNA gene (*rrn*) was used as a control. PCR products were separated in 1% agarose gel and stained with ethidium bromide. Also, RT-PCR was used to amplify the intergenic spaces between *bcr* genes to determine whether these genes are cotranscribed.

### Nucleotide Sequence Accession Number

Sequences of the *bcrD, bcrB, bcrA* and *bcrR* genes from *C. perfringens* were deposited. in GenBank database under accession numbers GU810179, GU810180 GU810181 and GU810182, respectively.

## Results

### *C. perfringens* Genotyping

Ninety-nine field isolates of *C. perfringens* were recovered from the caeca of 50 turkeys (81 isolates) and 13 chickens (18 isolates). Isolation rates of *C. perfringens* from caeca samples were 66% for turkeys and 30% for broilers. Of those 99 field isolates, genotyping results revealed that 96 were of type A (alpha toxin) and 3 were of type E (alpha and iota toxins). The three isolates associated with necrotic enteritis were of type A.

### Phenotypic Bacitracin Resistance

Of the 99 field isolates of *C. perfringens*, 24 of those showed bacitracin resistance (19 and 5 of turkey and chicken origin, respectively). The remaining 75 isolates were susceptible to bacitracin with MICs ranging from 0.75 µg/ml to 16 µg/ml (Table 1). The 24 resistant isolates demonstrated high-level bacitracin resistance by their elevated MIC results (>256 µg/ml). Percentages of *C. perfringens* isolates from chicken and turkeys that showed bacitracin resistance were 28% and 23.5%, respectively. MIC_90_ was calculated at >256 µg/ml for both poultry species. MIC_50_ was calculated at 6 µg/ml for turkey isolates and at 3 µg/ml for chicken isolates.

**Table 1:** MICs and percentages of bacitracin resistance of *C. perfringens* isolates.

Origin^(a)^	Number of isolates with a MIC (µg/ml) of										MIC _90_ b and MIC _50_ b (µg/ml)
	0.75	1	2	3	4	6	8	12	16	>256	
Turkey (81)	0	0	12	16	10	11	3	7	3	19	>256 and 6
Chicken (18)	2	1	5	1	1	1	1	1	0	5	>256 and 3

a Numbers of isolates; the three *C. perfringens* isolates of chicken origin associated with necrotic enteritis had MIC values of 3, 4 and 256 µg/ml; while the three type E isolates of turkey origin had MIC values of 4, 6 and 12 µg/ml.

b Concentration where growth was inhibited in 50% and 90% of the isolates.

The vertical line indicates the high level bacitracin resistance breakpoint of >256 µg/ml.

### MLVA Analysis

Bacitracin resistant strains (*n* = 24) were analyzed for their genetic relatedness using MLVA. The BioNumerics dendrogram yielded a total of 15 MLVA types for the 24 isolates. These types were separated in three clonal clusters, the first one containing the majority of the isolates (*n* = 16) (Fig. S1). The first cluster also contained all chicken isolates. Among the strains tested, there were 7 pairs of strains each one isolated from the same bird. Of those, only two had over 97% homology.

### Sequence Analysis

**Figure 1:**
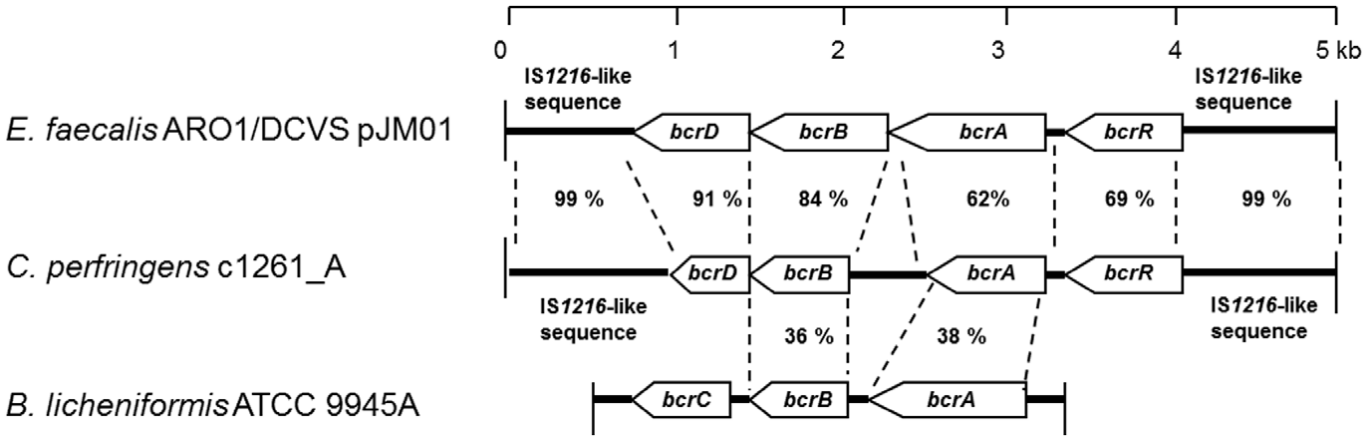
Comparison between the *C. perfringens bcrABDR* genes, the *E. faecalis bcrABD* operon and the *B. licheniformis bcrABC* operon. Organization of *C. perfringens* c1261_A resistance genes *bcrA, bcrB, bcrD,* and *bcrR* and comparison with the *bcrABD* operon of *E. faecalis* ARO1/DCVS (AY496968) and the *bcrABC* operon of *B. licheniformis* ATCC 9945A (L20573). Open arrows indicate ORFs. The amino acid percentages indicated relate to the identity between the amino acid sequences of the proteins encoded by the ORFs. The *bcrABC* operon of *B. licheniformis* is inverted to facilitate the comparison.

The high-level bacitracin resistant *C. perfringens* strain c1261_A with a MIC of >256 ug/ml and isolated from the caecum of a turkey was selected for sequencing analysis. Putative bacitracin resistance genes of this strain were sequenced using PCR amplification with walking primers that were designed based on the sequence of the *E. faecalis bcrABDR* operon. Analysis of the sequence data showed that the putative bacitracin resistance genes were contiguous on a DNA fragment of 4486 bp with four open reading frames (ORFs) that were oriented in the same direction and were designated *bcrR* (621 bp), *bcrA* (702 bp), *bcrB* (528 bp), and *bcrD* (654 bp) based on sequence identity with bacitracin resistance genes identified in *E. faecalis* [21] (Fig. 1). Using BLASTN, the complete DNA fragment of the *bcrABDR* genes of *C. perfringens* strain c1261_A showed 88% sequence identity to the *bcrABDR* genes previously described in *E. faecalis* plasmid pJM01 [21]. Upstream and downstream sequences (600 bp and 750 bp, respectively) exhibited high sequence identity (99%) to IS*1216*-like transposase gene of *E. faecalis* (Fig. 1).

The *bcrD* gene encodes for a putative 218 amino acid protein with an estimated molecular weight of 24 kDa. The amino acid sequence of BcrD was found to have 91% identity and 96% similarity to the BcrD protein in *E. faecalis* which is a putative undecaprenol kinase implicated in bacitracin resistance [44], [45]. Identities between 60% and 75% with undecaprenol pyrophosphate phosphatase of *Eubacterium ventriosum*, *Coprococcus eutactus*, and *Clostridium thermocellum* were also found for the BcrD protein of *C. perfringens* c1261_A. BcrB, a 176 amino acid putative protein with an estimated molecular weight of 19.8 kDa, shared 84% identity and 88% similarity to the *E. faecalis* BcrB protein with a membrane-spanning domain. BcrB also showed identity (between 35% and 40%) to a number of permeases associated with ABC transporters. BcrA is a 234 amino acid putative protein with an estimated molecular weight of 26.4 kDa. It has 62% identity and 72% similarity to the *E. faecalis* BcrA protein with an ATP-binding domain and also showed identity (between 40% and 45%) to other ATP binding proteins of ABC transporters. The complete amino acid sequences of BcrA and BcrB are predicted to be an homodimeric ABC transporter similar to the one recently described by Manson et al. (2004). The complete 207 amino acid sequence of BcrR contained a xenobiotic response element (XRE) conserved domain of the transcriptional regulators family from residue 2 to residue 55. BcrR shared 69% identity and 80% similarity to *E. faecalis* BcrR which is suspected to act as a sensor and a transducer of bacitracin availability [21]. Its molecular weight was estimated to be 23.6 kDa.

### PCR and Microarray

All 24 bacitracin resistant isolates were positive for the four *bcr* genes (*bcrABDR*) on both PCRs and microarrays. They were all also positive for *tetA(P),* which mediates active tetracycline efflux [46]; and for *tetB(P),* which is related to the ribosomal protection family of tetracycline-resistance determinants [46]. Only one isolate was positive for the *erm*(B) gene that encodes a ribosomal methylase that mediates MLS_B_ (macrolide-lincosamide-streptogramin B) resistance [47]. *C. perfringens* ATCC 13124 was negative for all the DNA probes. Plasmid curing experiments revealed the lost of the *tet* genes by PCR, indicating plasmid localization of the *tet* genes. However, in no cases, the bacitracin resistance genes proved to be curable. Also, to positively correlate the absence of a complete *bcr* locus with phenotypic susceptibility to bacitracin, 70 susceptible *C. perfringens* strains of this study were analyzed for the presence of *bcrABDR* genes and all strains were negative for all *bcr* genes.

### Efflux Pump Inhibitors

Of all efflux pump inhibitors tested, only thioridazine increased significantly the susceptibility of strain c1261_A to bacitracin (Table 2). The MIC of this strain moved from 512 µg/ml to 8 µg/ml with increasing concentrations of thioridazine which resulted in a bacitracin susceptible phenotype. The MIC was only slightly reduced with MK-571 (512 to 128 µg/ml), verapamil (512 to 128 µg/ml), prochlorperazine (512 to 256 µg/ml) and reserpine (512 to 256 µg/ml). Probenecid did not show any influence on the MIC of strain c1261_A (512 µg/ml throughout the experiments). The same results were obtained in all three independent checkerboard assays.

**Table 2:** Bacitracin MIC values (µg/mL) of *C. perfringens* strain c1261_A grown with different concentrations of efflux pump inhibitors.

	Efflux pump inhibitor concentration (µg/mL)												
	0	1	2	4	8	16	32	64	128	256	512	1024	2048
Efflux pump inhibitor(MIC, µg/mL)	Bacitracin MIC value (µg/mL)												
MK-571 (64)	512	512	512	512	512	256	128	–					
Thioridazine (16)	512	512	256	256	8	–	–	–					
Prochlorperazine (64)	512	512	512	512	512	512	256	–					
Verapamil (1024)	512	512	512	512	512	512	512	512	512	256	128	–	–
Probenecid (1024)	512	512	512	512	512	512	512	512	512	512	512	–	–
Reserpine (2048)	512	512	512	512	512	512	512	512	512	512	512	256	–

Blank area, concentrations not tested; –, no bacterial growth due to efflux pump inhibitors.

### Semi-quantitative RT-PCR and Amplification of the Intergenic Spaces Between *bcr* Genes

**Figure 2:**
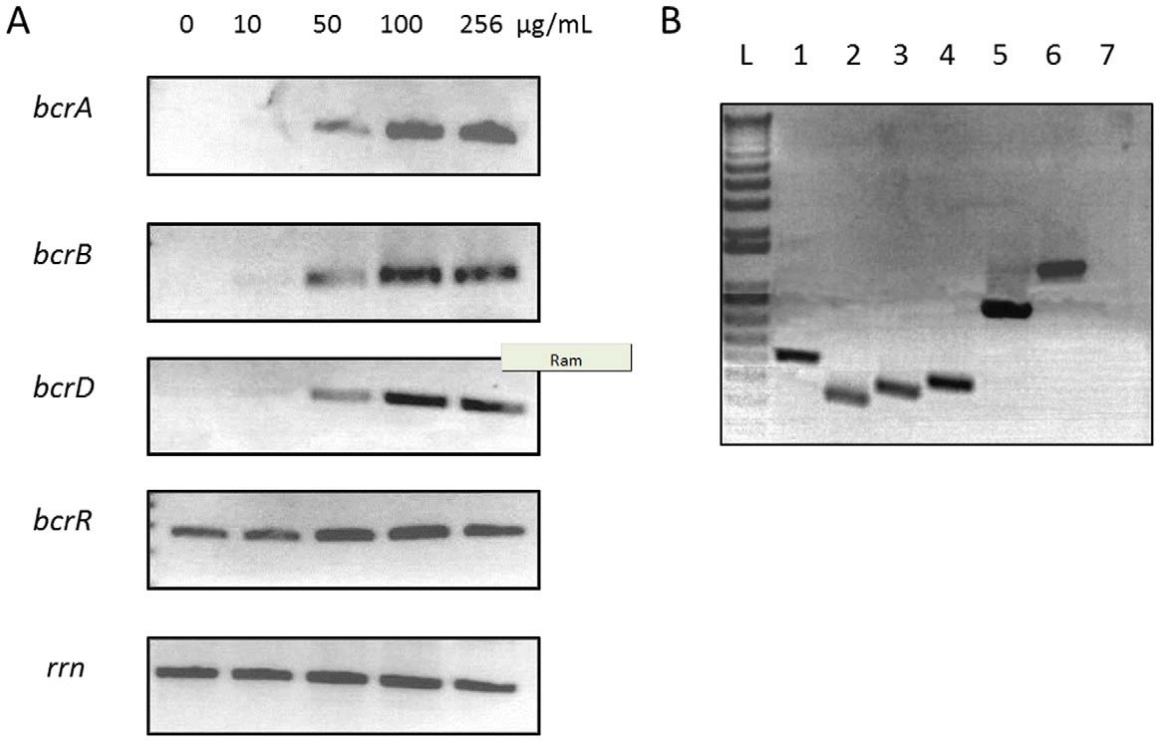
Expression and cotranscription of *bcrABDR* genes in presence of bacitracin. A) Semi-quantitative RT-PCR analysis of strain c1261_A grown in various concentrations of bacitracin. Expression of 16S rRNA gene (*rrn*) was used as a control. B) Amplification of intergenic regions by RT-PCR of strain c1261_A grown in presence of 256 µg/mL of bacitracin. Lane L, DNA ladder; Lane 1, *bcrA*; Lane 2, *bcrB*; Lane 3, *bcrD*; Lane 4, *bcrR*; Lane 5, *bcrD*/*bcrB* intergenic region; Lane 6, *bcrB*/*bcrA* intergenic region; Lane 7, *bcrA*/*bcrR* intergenic region.

To investigate the expression of *bcrABDR* gene cluster in presence of bacitracin, mRNA levels were analysed by semi-quantitative RT–PCR. Data showed negligible or no *bcrABD* mRNA in absence of bacitracin (Fig. 2A). When bacitracin was present in the growth medium, the levels of the *bcrABD* transcripts increased as the concentration of bacitracin in which the cells were grown increased. For the *bcrR* gene, the analysis showed that it was expressed constitutively. Also, RT-PCR was used to amplify the intergenic spaces between *bcr* genes to determine whether these genes are cotranscribed (Fig. 2B). Results indicated that the *bcrABD* genes are transcribed as a polycistronic message and thus are part of an operon.

### Genomic DNAs PFGE, Plasmid and Hybridization Analysis

**Figure 3:**
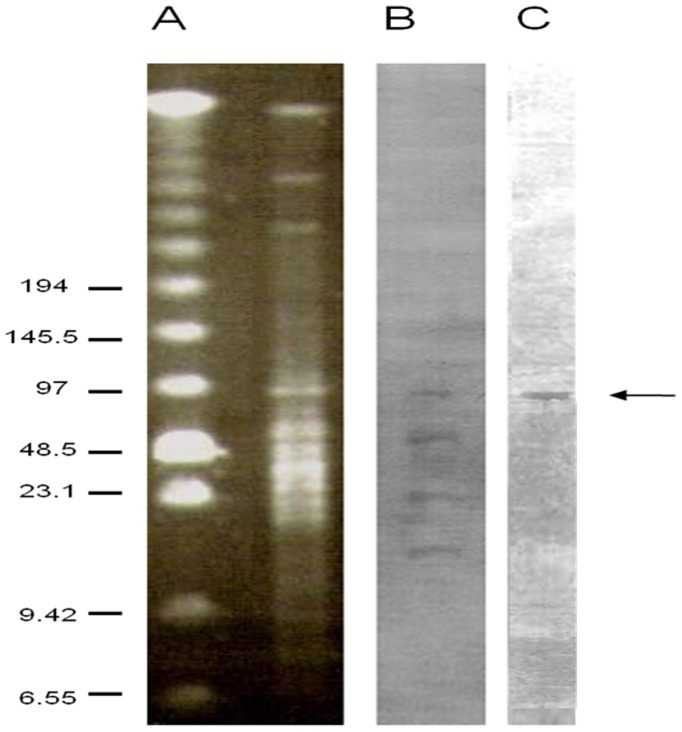
PFGE and hybridization analysis of I-CeuI and MluI double-digested DNA of the bacitracin resistant *C. perfringens* strain c1261_A. PFGE analysis of *C. perfringens* strain c1261_A total DNA (A). Southern blot of *C. perfringens* isolate c1261_A total DNA probed with *rrn* (B) and with *bcrB* (C). Sizes (in kilobases) are indicated on the left.

Plasmid extraction and analysis of *C. perfringens* isolate c1261_A showed five bands with molecular weights of approximately 3.5 kb, 4 kb, 7.5 kb, 11.5 kb, and 23 kb (data not shown). None of these bands hybridized with the *bcrB* probe. I-CeuI and MluI double-digested genomic DNA of the bacitracin resistant *C. perfringens* strain c1261_A hybridization experiments with *bcrB* and *rrn* probes showed cohybridization of these probes on one band corresponding to a ∼97 kb chromosomal fragment (Fig. 3). Control experiments showed no cross-hybridization between probes (data not shown).

## Discussion

While phenotypic bacitracin resistance has been reported in the past in *C. perfringens*, the genetic basis behind this resistance has not been determined [26], [28]. A collection of *C. perfringens* isolates originating from turkey and chicken intestinal tracts was set up and each isolate was typed before susceptibility to bacitracin was determined. Ninety-nine field isolates of *C. perfringens* were recovered from the caeca of turkeys (81) and chickens (18). The *C. perfringens* isolation rates from caecal samples were higher for turkeys (66%) than for broiler chickens (30%). This is in agreement with a previous study [28] which demonstrated that *C. perfringens* isolation was more difficult after the chickens reached 22 days of age due to modifications of their intestinal flora. Some birds might have been fed with a bacitracin supplemented diet that could have contributed to a lower recovery rate, as it has been found that bacitracin reduces the count of *C. perfringens* in the caeca of the treated birds [48]. Most of the isolates recovered in the present study were of type A, which is also concordant with previously published data [28], [49].

To elucidate the genetic basis of bacitracin resistance in *C. perfringens*, the high-level bacitracin resistant (MIC >256 µg/ml) *C. perfringens* strain c1261_A was selected for further studies using a primer walking strategy based on an enterococcal *bcrABD* operon. Sequence analyses of the amplicons generated by the *bcr* primers showed four contiguous ORFs. Two of these genes had high levels of amino acid sequence identity to the *bcrA* and *bcrB* genes of *E. faecalis* suggesting that the genes identified in this study encode for an ABC transporter. This is further supported by the high-level bacitracin resistance observed in the *bcrABDR* positive strains of *C. perfringens*. Reports in the literature suggest that the level of bacitracin resistance given by the ABC transporter is higher than those produced by any other mechanism [45], [50]. Matos et al. (2009) recently reported that the *bcrA* and *bcrB* genes of *E. faecalis* were sufficient for high-level bacitracin resistance [25].

In addition, *C. perfringens* strain c1261_A was exposed to efflux pump inhibitors to investigate their influence on susceptibility to bacitracin. Drug efflux pumps belonging to the ABC, MATE (multiple antimicrobial and toxin extrusion), MFS and SMR (small multi-drug resistance) families are found in Gram-positive bacteria, with MFS-class pumps being predominant [51]. Inhibition of these pumps has been shown to reverse acquired resistance [51]. Some bacterial efflux pump inhibitors have been proposed as viable strategies to combat increasing antimicrobial resistance [51]. Thioridazine is a general bacterial multidrug efflux pump inhibitor which creates ultrastructural changes in membrane-bound enzymes [52] Verapamil, reserpine and prochlorperazine target the P-glycoprotein-mediated efflux mechanism [51] while probenecid and MK-571 have inhibitory activity against the multidrug resistance-associated protein (MRP) transporters [53], [54]. Probenecid has also an activity on organic anions transporters [54]. Results with verapamil, reserpine, prochlorperazine and MK-571 compounds showed a minimal inhibitory effect with a slight influence on the bacitracin MIC values; while probenecid did not influence the MIC of bacitracin. Since thioridazine was the only inhibitor that significantly increased the susceptibility of strain c1261_A to bacitracin, it can be assumed that an efflux pump is involved in bacitracin resistance and that this mechanism is likely organic anions transporters-independent. Also, it can be speculated that MRP and P-glycoprotein mediated efflux are not the main mechanisms involved in bacitracin resistance. Definitive functional proves that the *bcrABDR* genes can confer bacitracin resistance in these strains awaits construction and complementation of deletion mutants (*bcrR*, *bcrAB*, *bcrRAB, bcrD*, *bcrRD)* which is presently underway in our laboratory. Semi-quantitative RT-PCR demonstrated that the *bcrABDR* gene cluster expresses when *C. perfringens* are subjected to bacitracin stress. It was also found that the *bcrABD* genes are transcribed as a polycistronic message and thus are part of an operon. These results are in agreement with the results obtained by Manson *et al* [21], who found that the ABC transporter and an overproduced undecaprenol kinase were both encoded by a *bcrABD* operon under the control of a regulator *bcr*R, which was described as a membrane bound sensor and a transducer of bacitracin availability to regulate *bcrABD* expression.

Plasmid and hybridization analyses revealed that the location of the *bcr* genes of the *C. perfringens* c1261_A strain was not on plasmid fragments. To further explore the *bcr* genes location, PFGE was performed with chromosomal DNAs. The I-CeuI restriction enzyme that was used cuts only in the 23S rRNA genes (*rrn* genes), which are localized only on the chromosome. Thus, chromosomal fragments could be differentiated from plasmid bands with the *rrn* probe. In this study, both the *bcrB* and *rrn* probes colocalized on a ∼97 kb fragment indicating that the *bcr* are chromosomal genes. This is in agreement with previous reports where all other bacterial genera, except for *E. faecalis* [21], were harbouring bacitracin resistance genes on the chromosome [24], [50], [55]. Curiously, the putative ABC transporter identified in this study seems genetically closer to the acquired plasmid-borne ABC transporter in *E. faecalis* [21] than the ABC transporters previously described for *B. licheniformis*, *B. subtilis* and *S. mutans* [24], [50], [55]. Also, it is potentially regulated by a single protein which contains a XRE domain with homology to the *E. faecalis* regulator, BcrR [21]. The XRE family of transcriptional regulators contains also regulators involved in stress responses in bacteria [56]. In all other bacitracin efflux ABC transporters described to date, such as *B. licheniformis* and *B. subtilis*, regulation is performed by a two-component system of a sensor kinase and a response regulator localized on a chromosomal operon. There are also some differences between the *bcrABDR* cluster of this study and the one described in *E. faecalis*. The *C. perfringens bcrD*, *bcrB* and *bcrA* are smaller than their *E. faecalis* counterparts (185 bp, 226 bp, and 234 bp shorter, respectively). The distance between the *bcrA* and the *bcrB* genes is larger in *C. perfringens* (441 bp versus -7 bp in *E. faecalis*). We also report, for the first time to our knowledge, on the insertion of IS*1216*-like transposase gene flanking the chromosomal bacitracin genes of *C. perfringens* c1261_A. These results suggest that this IS*1216*-like element originated from *E. faecalis* and that we may anticipate the emergence of novel, IS*1216*-based composite mobile elements in *C. perfringens*.

Microarray results revealed that the bacitracin resistant strains of this study all contained both *tetA(P)* and *tetB(P)* genes, which mediate active tetracycline efflux and ribosomal protection, respectively [46], while only one isolate harbour a MLS_B_ resistant gene, *erm*(B). This is in agreement with previous studies [27], [57] that reported on reduced susceptibility to tetracycline in poultry *C. perfringens*. However, no other antimicrobial resistant genes were detected in our isolates indicating a low degree of resistance to most other antimicrobials tested as previously described [27], [57].

In conclusion, this study reports for the first time the characterization of putative bacitracin efflux pump and an overproduced undercaprenol kinase genes associated with acquired bacitracin resistance in a high-level bacitracin resistant *C. perfringens* strain c1261_A of poultry origin. Zinc bacitracin is used in poultry production in the United-States and Canada. This practice has likely selected for *C. perfringens* strains encoding for bacitracin resistance genes. Bacitracin resistance genes have been described in *E. faecalis* and these genes have high sequence identities with the genes described in this study; thus, a common origin for the bacitracin resistance genes between the two genera is possible. Further investigations are needed to determine the role of each gene and how widespread these genes are in *Clostridium* strains and species from different origins.

## Supporting Information

Figure S1 **Dendrogram of the MLVA types of** ***C. perfringens*** **resistant isolates.** Cluster analysis was performed with UPGMA using Pearson coefficient. P: chicken strains; the remaining isolates are of turkey origin.
(TIF)

Table S1 **Primers for sequencing novel bacitracin resistant genes of** ***C. perfringens*** **strain c1261_A using the primer walking method and new primer designs for PCR screening of** ***bcrA*** **,** ***bcrB*** **,** ***bcrD*** **and** ***bcrR*** **.**
(DOC)

Table S2 **New probe sequences added to the antimicrobial resistance microarray^a^.** ^a^ The *bcrR* probe sequence was already on the array. ^b^ *ermQ,* a ribosomal methylase gene that mediates MLS_B_ resistance [40]; *bcrABD* genes, ABC transporter genes (our study); *tetB(P)*, tetracycline ribosomal protection protein gene [39]; other genes of the antimicrobial resistance microarray were previously described [33].
(DOCX)
