# Supplementary figures and images for: Characterization of Genes Encoding for Acquired Bacitracin Resistance in Clostridium perfringens
Source: PLoS One. 2012 Sep 6;7(9):e44449. doi: 10.1371/journal.pone.0044449 (PMC3435297; doi:10.1371/journal.pone.0044449)

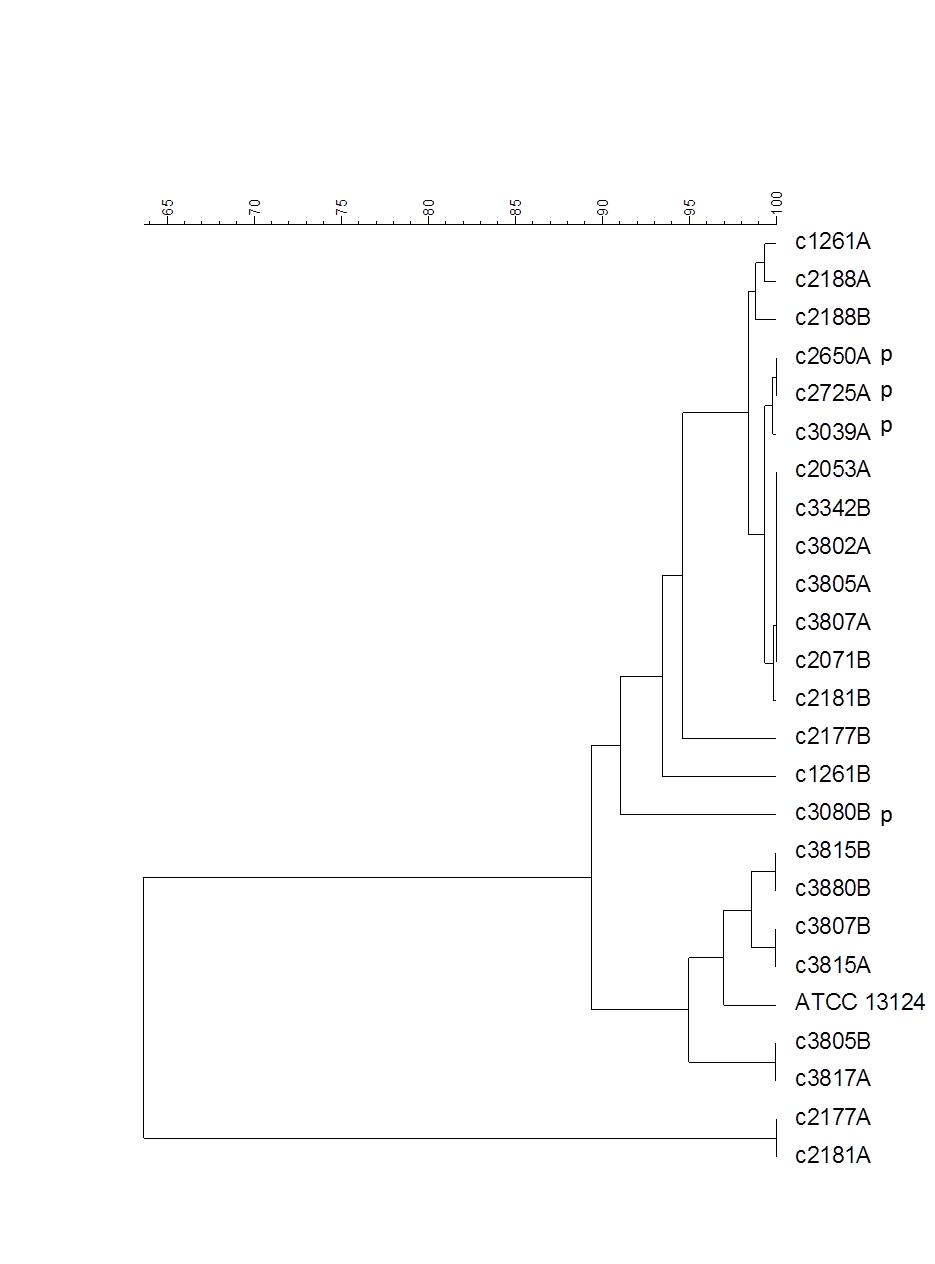

Supplement: Figure S1 — Dendrogram of the MLVA types of C. perfringens resistant isolates. Cluster analysis was performed with UPGMA using Pearson coefficient. P: chicken strains; the remaining isolates are of turkey origin. (TIF) [file pone.0044449.s001.tif]
