# Supplementary material for: Characterization of Genes Encoding for Acquired Bacitracin Resistance in Clostridium perfringens
Source: PLoS One. 2012 Sep 6;7(9):e44449. doi: 10.1371/journal.pone.0044449 (PMC3435297; doi:10.1371/journal.pone.0044449)
Supplement: Table S1 — Primers for sequencing novel bacitracin resistant genes of C. perfringens strain c1261_A using the primer walking method and new primer designs for PCR screening of bcrA , bcrB , bcrD and bcrR . (DOC) [file pone.0044449.s002.doc]

Table S1. Primers for sequencing novel bacitracin resistant genes of *C. perfringens* strain c1261_A using the primer walking method and new primer designs for PCR screening of *bcrA*, *bcrB*, *bcrD* and *bcrR*.

| **Primer for sequencing** | **Primer sequence**  **5’ – 3’** | **Location** | **Size of product (bp)** |
| --- | --- | --- | --- |
| 1  Forward  Reverse | TACCGTTGGGTGCAAGAATACGGA  ATAGGAGTTTCACGAGTGGCAGCA | 26-60  1031-1055 | 1030 |
| 2  Forward  Reverse | CCATGCCAATGACAAGTGTGAGCA  AGGGCATATGCTTCTTGTGGACGA | 941-965  1459-1483 | 543 |
| 3  Forward  Reverse | ACACAAGCTACCGCAACCTTGAAC  TCTGCCGTGATTGTCATGGGAAGT | 1341-1364  1750-1774 | 434 |
| *bcrB*  Forward  Reverse | AAAGAAACCGACTGCTGATA  GCTTACTTGTATAGCAGAGA | 1622-1641  2091-2110 | 489 |
| 4  Forward  Reverse | ACAGCGTCATATAGCCCACAAACG  TCAAACTGATTGGAGGTGGCGGT | 2016-2040  2331-2355 | 340 |
| 5  Forward  Reverse | CAGTACGCTGTCGCTGTATA  CTGACACGATAAGTGTCGGA | 2156-2175  3368-3387 | 1232 |
| 6  Forward  Reverse | CCGTTTCTGCCGAGCAAACCATAA  GCACAAGGGATAGCGAAACTGATG | 3213-3237  3496-3520 | 308 |
| *bcrR*  Forward  Reverse | TATAGGGTTCTCTTGCCGCT  GTTACCCTAACATGGAGTCG | 3488-3507  3894-3913 | 426 |
| 7  Forward  Reverse | AGTTCTTCGCCCGATAGCAGTCAT  GGGCATATCGGAAAGACGGATACA | 3905-3929  4156-4180 | 276 |
| 8  Forward  Reverse | GTAACCCTTGCCGCTTTCCCATTT  AGAACCCATAATAGAGCGTGGCGT | 3987-4011  4488-4512 | 526 |
| **PCR Primer** | **Sequence (5’-3’)** | **Product length (bp)** | |
| *bcrA*_CP-F | GGCAATACCAAGCCGTTGCTTCAT | 408 | |
| *bcrA*_CP-R | TTACGAAGCGATACGGAACAGCCA |
| *bcrB*_CP-F | ATAGGTGGCTGTCCACGGATACAA | 247 | |
| *bcrB*_CP-R | CGTTTGTGGGCTATATGACGCTGT |
| *bcrD*_CP-F | TGCTGCCACTCGTGAAACTCCTAT | 318 | |
| *bcrD*_CP-R | TTCTCGTTGTGGTTCAAGGTTGCG |
| *bcrR*_CP-F | AAGGGCGGTTACATAGGGTTGTCT | 379 | |
| *bcrR*_CP-R | ATGTCTGCTATCGGGCGAAGAACT |
