# Supplementary material for: Characterization of Genes Encoding for Acquired Bacitracin Resistance in Clostridium perfringens
Source: PLoS One. 2012 Sep 6;7(9):e44449. doi: 10.1371/journal.pone.0044449 (PMC3435297; doi:10.1371/journal.pone.0044449)
Supplement: Table S2 — New probe sequences added to the antimicrobial resistance microarraya. a The bcrR probe sequence was already on the array. b ermQ, a ribosomal methylase gene that mediates MLSB resistance [40]; bcrABD genes, ABC transporter genes (our study); tetB(P), tetracycline ribosomal protection protein gene [39]; other genes of the antimicrobial resistance microarray were previously described [33]. (DOCX) [file pone.0044449.s003.docx]

Table S2. New probe sequences added to the antimicrobial resistance microarray^a^.

| **Gene^b^** | **Microarray probe sequence** | | |  |  |  |  |
| --- | --- | --- | --- | --- | --- | --- | --- |
|  |  |  | |  |  |  |  |
| *bcrA* | gcttgtttggcacaaactataaggaaaatatccataccctttatagcaaagtaggctctattatcgaaac | | | | | | |
|  |  |  |  | |  |  |  |
| *bcrB* | cctgcttctttggattgttatgctcacccttgtaacatgggcaggcatatttatcgtttgtgggctatat | | | | | | |
|  |  |  |  | |  |  |  |
| *bcrD* | ttggaacaagatgtttccctttcaatttaagaacaaagcacagtcaattgttaaaaaggatactttctcg | | | | | | |
|  |  |  |  | |  |  |  |
| *tetB(P)* | ttaatctctaattgcgtttagtttatgaagcaaatactcttttttattcaatggatcatacgttgttttt | | | | | | |
|  |  |  |  | |  |  |  |
| *ermQ* | cctaggtatttaattactgcatgaatttgattttttgtaaatacatctcttaagttattaatagacttag | | | | | | |
